# Shades of Brown: A Model for Thermogenic Fat

**DOI:** 10.3389/fendo.2015.00071

**Published:** 2015-05-08

**Authors:** Jon Dempersmier, Hei Sook Sul

**Affiliations:** ^1^Comparative Biochemistry Program, Department of Nutritional Science and Toxicology, University of California Berkeley, Berkeley, CA, USA

**Keywords:** Brown fat, Brown adipose tissue, beige/brite adipocytes, nonshivering thermogenesis, Zfp516

## Abstract

Brown adipose tissue (BAT) is specialized to burn fuels to perform thermogenesis in defense of body temperature against cold. Recent discovery of metabolically active and relevant amounts of BAT in adult humans have made it a potentially attractive target for development of anti-obesity therapeutics. There are two types of brown adipocytes: classical brown adipocytes and brown adipocyte-like cells, so-called beige/brite cells, which arise in white adipose tissue in response to cold and hormonal stimuli. These cells may derive from distinct origins, and while functionally similar, have different gene signatures. Here, we highlight recent advances in the understanding of brown and beige/brite adipocytes as well as transcriptional regulation for development and function of murine brown and beige/brite adipocytes focusing on EBF2, IRF4, and ZFP516, in addition to PRDM16 as a coregulator. We also discuss hormonal regulation of brown and beige/brite adipocytes including several factors secreted from various tissues, including BMP7, FGF21, and irisin, as well as those from BAT itself, such as Nrg4 and adenosine.

## Introduction

The pandemic rise in the prevalence of obesity, as well as its comorbidities, such as type-2 diabetes and cardiovascular diseases, has made it imperative to develop novel anti-obesity therapeutics. Obesity results from chronic excess of energy consumed through the diet versus energy expenditure through basal metabolism, physical exertion, and adaptive thermogenesis. Thus, successful therapies must involve either increasing energy expenditure or preventing absorption of calories from the diet, both of which have been used to limited success. One area of relative promise centers on targeting brown adipose tissue (BAT) to increase energy expenditure. Differing from white adipose tissue (WAT), which stores excess energy from the diet as triglycerides, BAT is a thermogenic organ, metabolizing both fatty acids and glucose to produce heat to maintain homeothermy. The thermogenic activity of BAT is possible due to the presence of a BAT-specific mitochondrial proton transport protein, uncoupling protein 1 (UCP1), which uncouples the mitochondrial proton gradient from ATP synthesis and generates heat. While existence of BAT in infants as well as discrete populations of adult humans had been shown many years ago (1), recent findings using ^18^fluoro 2-deoxyglucose positron emission tomography (FDG-PET) verified the presence of metabolically active BAT in human adults (2, 3). Thus, developing BAT may provide an attractive new target for prevention/treatment of obesity. A better understanding of BAT development and function has the potential to improve the health of millions of Americans and others affected by obesity and its associated disorders.

## Thermogenesis: A Sum of all BATs

Earliest work on BAT centered on its function in thermogenesis, identifying the basic tenets of BAT activation in response to cold. Upon cold exposure, sympathetic nervous system (SNS) innervating BAT releases norepinephrine, which, via β_3_-adrenergic receptor (AR)-cAMP-PKA pathway, stimulates lipolysis to produce fatty acids for oxidation. These fatty acids can also allosterically activate UCP1-mediated uncoupling, as UCP1 functions as a H^+^/fatty acid symporter (4, 5). Furthermore, via CREB/ATF2, stimulation of β_3_-AR induces BAT-enriched genes, including UCP1 (6). β_3_-AR stimulation also increases expression of PGC1α, which is critical for mitochondrial biogenesis to support BAT thermogenesis.

Classical brown adipocytes come from a developmental lineage distinct from WAT, arising from Myf5^+^/Sca-1^+^/Pax7^+^ cells of the dermomyotome, a common progenitor of skeletal muscle and dermis (7–9). Following treatment with bone morphogenic protein-7 (BMP7), these precursor cells can be induced to differentiate into brown adipocytes (10). While many transcriptional and hormonal regulators of brown adipocyte differentiation and thermogenesis have been identified as discussed below, commitment to brown preadipocytes from multipotent precursors is yet to be clearly defined.

As our understanding of BAT development and function has progressed, it became apparent that non-shivering thermogenesis of BAT is only half of the coin. The earliest reports of the presence of brown adipocyte-like cells in WAT depots in mice following cold acclimation stretch back over 30 years (11). Indeed, various treatments have been shown to result in the rise of UCP1^+^ cells in WAT, but the physiological relevance of these cells has been poorly understood. However, seminal work by Schulz et al. showed that severing sympathetic neurons to brown fat and blocking BAT development both lead to compensatory rise in brown-like adipocytes in WAT (12). Thus, WAT depots can act as reserve thermogenic pool of cells for cold adaptation, although the overall contribution to whole-body thermogenesis is not known. The ability for cells within a WAT depot to give rise to UCP1^+^ cells varies depot by depot, as well as with different strains of mice. With chronic cold exposure, all depots are capable of undergoing some degree of browning (13). Thus, total thermogenic capacity for mice might be dependent on the contribution of all UCP1^+^ cells in all adipose organs rather than BAT alone.

Recent characterization of the brown adipocyte-like cells in WAT depots has revealed them to be distinct from classic brown adipocytes. These so-called beige or brite cells (brown-in-white) have low expression levels of UCP1 at ambient temperature, but are greatly induced upon cold exposure. The process of ‘browning’ or recruitment is primarily under sympathetic control and ablation of β3-AR has been reported to severely block formation of beige/brite adipocytes in WAT depots. The browning process, however, is one of much debate as to whether cold challenge leads to recruitment of preadipocytes that are distinct from white precursors to differentiate into UCP1^+^ adipocytes, or whether white adipocytes present in the tissue are interconverted to become UCP1^+^ cells. Indeed, early work by Himms-Hagen et al. showed that following 7-day-treatment of the β3 agonist, CL-316,243, the majority of multilocular cells, indicative of beige/brite adipocytes, were negative for BrdU staining, suggesting interconversion as the primary pathway for induction of UCP1^+^ cells (14). However, using pulse-chase experiments employing the adiponectin-based AdipoChaser model, Wang et al. recently showed that adipocytes in subcutaneous or inguinal WAT (iWAT), that were indelibly labeled with LacZ prior to cold exposure, did not express UCP1 following 3 days of cold exposure. Thus, UCP1 expression was confined to LacZ^−^ cells, suggesting *de novo* differentiation of distinct precursor cell populations (15). Interestingly, this phenomenon was limited to iWAT. In epididymal WAT (eWAT), cold exposure led to *de novo* adipogenesis without UCP1 expression. This is contradictory to the observation that cold acclimation led to substantial browning of almost all WAT depots (13, 16). On the other hand, Lee et al. reported that, while the majority of browning of iWAT resulted from recruitment of preadipocytes to become UCP1^+^ adipocytes, thermogenic adipocytes in eWAT arose from interconversion from white adipocytes (17). Could there be a depot-specific mechanism for browning or is there a common intermediate for thermogenic cells? Perhaps, the differences in adrenergic innervation of the two depots may lead to a requirement for noradrenergic fiber branching prior to recruitment of beige/brite adipocytes in eWAT (18). Interestingly, Rosenwald et al. showed that recruited beige/brite adipocytes were able to revert back to white adipocytes once the stimulus was removed. When restimulated, these same cells were converted back again to thermogenic beige/brite adipocytes (19). The authors also showed that upon restimulation, a percentage of the cells positive for UCP1 were not labeled upon the original cold stimulation, potentially signifying the recruitment of new beige/brite adipocytes. Whether these adipocytes are recruited from progenitor populations signifying *de novo* recruitment or indicative of a limitation of the system is unclear. Regardless, the ability for beige/brite cells to interconvert to white adipocytes makes discerning the mechanism for browning difficult.

In an attempt to identify precursors of the brown-like cells in WAT, Wu et al. performed clonal expansion of preadipocytes of iWAT and identified non-thermogenic and thermogenic precursor populations each with unique gene expression signatures. The thermogenic lines expressed many common genes with those of BAT (UCP1, PGC1α) as well as beige-specific genes such as Tmem26, Tbx1, CD137, and CD40 (20). Interestingly, CD40 ligand (CD40L) in humans has been correlated with obesity (21). Disruption of CD40 signaling in mice has been reported to increase UCP1 expression in BAT and to protect against diet-induced obesity (22). With this contradictory observation, the beige markers so far identified need to be further characterized and verified. Further complicating the matter, Lee et al. isolated PDGFRα^+^ cells from eWAT and found them to be bipotent precursors that could differentiate into either brown or white adipocytes *in vitro*. However, transplants of these cells into mice resulted in only UCP1^−^ white adipocytes, even after CL-316,243 treatment (17).

**Figure 1:**
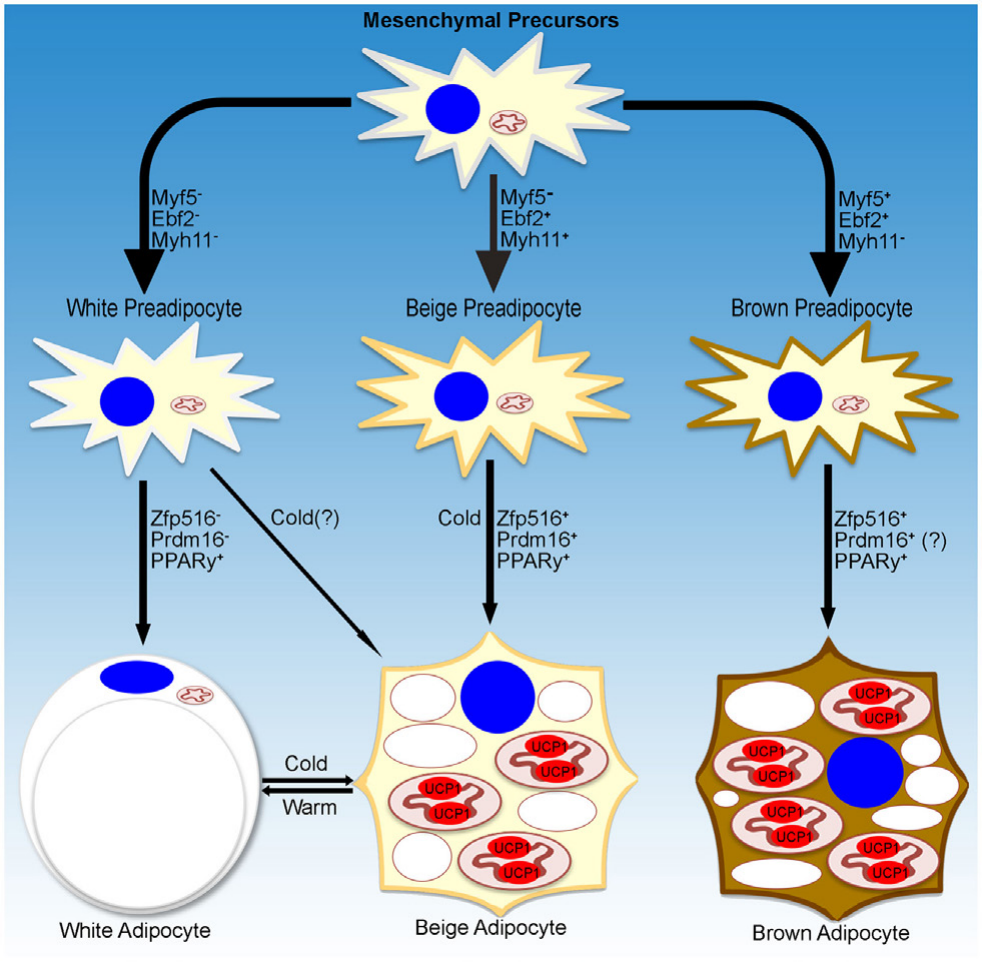
**Transcriptional regulation of adipocyte development**. Schematic representation of markers denoting specific lineages of thermogenic adipocytes. Transcriptional regulators and environmental cues for adipocyte development discussed in the text are listed as well.

While a small subset of cells in WAT depots have been reported to be Myf5^+^, recent studies have shown that these cells had a reduced capacity to become brown-like adipocytes (23, 24). On the other hand, using an UCP1-promoter reporter system, Long et al. identified a population of beige/brite adipocytes that arose from cells that are positive for Myh11, a smooth muscle-like marker. However, within the iWAT depot, not all of the UCP1^+^ cells were Myh11^+^, suggesting that, even within a single depot, heterogeneity of beige cells may exist. Further studies are necessary to define whether beige adipocytes are a distinct population of cells within WAT depots and whether these cells arise from distinct precursors, and if they do, to define the lineage of these cells (Figure 1).

## Human BAT: Brown, Beige, or Shades of Brown

As mentioned above, the classic dogma in human BAT centers around the previously accepted fact that interscapular BAT, anatomically similar to that in rodents, is abundant in infants but decreases with age (25). However, reports of detectable amounts in young, lean adults following cold stimulation do exist (26). Recently, prospective studies using FDG-PET scanning identified UCP1^+^ BAT in a majority of adults in the cervical, supraclavicular, and paravertebral areas, which was responsive to cold and negatively regulated by warm environment, sympathetic blockade, or dietary intervention (2, 27, 28). These findings showed a great therapeutic potential, as many groups have shown that increased BAT activity and function were protective against diet-induced obesity and insulin resistance in mice. Moreover, recent human studies showed similar observations. Cold exposure in humans led up to a 12-fold increase in glucose uptake, an increase in energy expenditure, and improved insulin sensitivity (29, 30). The caveat to these studies is that the beneficial effects of cold exposure were based on having measureable amounts of human BAT. Yoneshiro et al. reported that spending 2 h/day at 17°C for 6 weeks resulted in significant recruitment of BAT in the majority of subjects, leading to an increase in cold-induced thermogenesis and a decrease in body fat mass, which was proportional to BAT activity (31). Cooling subjects for extended periods of time below thermoneutrality, but not to the point of shivering, showed the prevalence of BAT to be close to 100% and thus individualized cold training could be a viable anti-obesity therapeutic (32). However, therapeutic use of β-agonists or other sympathomimetics could not be used due to either inefficacy or cardiovascular side effects, including increased risk of cardiac events (33–35). More research is needed to identify new therapeutics for increasing BAT recruitment and activity.

Understanding the nature of human BAT and whether it resembles classic murine brown or beige/brite cells is critical for therapeutics development, and is the subject of debate (36). Early work on this issue using BAT depots identified by FGD-PET (20, 37) showed similar results that human BAT expresses both TMEM26 and CD137, murine beige/brite markers, but not LHX8 or ZIC1, iBAT selective markers. Interestingly, later work by Jespersen et al. on supraclavicular biopsies found elevated expression of LHX8 and ZIC1, as well as TMEM26 and CD137, suggesting that both beige/brite and brown adipocytes may be present. Cypess et al. profiled adipose tissue from five different sites in patients found that deep neck fat expressed high levels of iBAT markers, ZIC1and LHX8, as well as UCP1, while superficial fat depots expressed WAT-selective HOXC9 and leptin. Interestingly, all depots, including iBAT, were found to express the beige markers, TMEM26 and CD137 (3, 25). These data suggest that TMEM26 and CD137 may be better described as pan-thermogenic adipocyte markers in humans, rather than beige/brite markers. Regardless, the presence of ZIC1^+^/LHX8^+^ depots suggests that adult humans have both beige/brite and brown adipocytes and thus both tissues may have therapeutic importance. However, the role of ZIC1 and LHX8 in thermogenesis is largely unexplored and thus, expression of these genes may not constitute a functional difference between beige and brown adipocytes.

## Transcriptional Regulation of Brown Adipogenesis

Numerous transcription factors, metabolites, and signaling molecules have been implicated in activating brown adipocytes and/or brown-fat differentiation (38, 39). Here, we highlight several recently identified key players in BAT gene expression and development, and regulation of non-shivering thermogenesis.

Earliest work on identifying critical transcriptional regulators of thermogenesis centered on the regulation of the UCP1 promoter. Sympathetic regulation of UCP1 in response to cold has been defined to be mediated by βAR–cAMP–PKA through CREB/ATF2 via p38 (40, 41). Both CREB and ATF2 could bind to the cAMP response element (CRE) at the proximal UCP1 promoter as well as at the conserved DNase I hypersensitive enhancer region at −2.5 kb upstream of the transcription start site (42, 43). This enhancer region also contains common response elements, including thyroid hormone response element (TRE), peroxisome proliferator-activated receptor response element (PPRE), as well as retinoic acid response element (RARE). However, all of the factors that can bind to these elements are somewhat ubiquitous and thus do not easily explain BAT-specific and sympathetically regulated expression of UCP1.

One of the first to be reported as a BAT-enriched regulator of thermogenesis is PR-domain containing protein 16 (PRDM16). PRDM16 has been shown to function as a coactivator of brown adipogenesis through interaction with several transcription factors, including C/EBPβ, CtBPs, PGC1α, and PPARγ, as well as the H3K9 histone methyltransferase, EHMT1, to drive brown fat/muscle precursors toward a brown-fat lineage (44–46). Indeed, in fibroblasts and C2C12 myoblasts, ectopic expression of PRDM16 and C/EBPβ was sufficient to drive a brown-fat transcription program leading to functional brown adipocytes (47). However, conditional deletion of PRDM16 in two different mouse models using adiponectin-Cre or Myf5-Cre found PRDM16 to be dispensable for BAT development. However, these mice showed defects in BAT maintenance during aging. In addition, ablation of PRDM16 by adiponectin-Cre in WAT blocked recruitment of beige adipocytes demonstrating a role of PRDM16 in browning of WAT (48, 49). More work is needed to better understand the role of PRDM16 in BAT development and browning of WAT.

In an attempt to identify brown fat-specific PPARγ-binding sites, Rajakumari et al. performed ChIP-seq on PPARγ and found that PPARγ sites in BAT coincided with early B-cell factor (EBF) binding sites, particularly within the UCP1 and PRDM16 promoters. They further showed that loss of EBF2 of the EBF family of transcription factors resulted in a complete loss of BAT characteristics, with little to no expression of UCP1, PRDM16, or CideA, while general adipogenic markers, such as PPARγ, were unaffected (50). Cell sorting for GFP^+^ cells in EBF2/GFP mice showed that EBF2 reliably marked preadipocytes of brown adipogenic potential present in both BAT and iWAT (51). Given the heterogeneity of beige cells within tissues, it would be interesting to see if all UCP1^+^ cells in various WAT depots are derived from EBF2^+^ cells.

Cold-inducible regulation of UCP1 was thought to be, along with CREB/ATF2, through the participation of PGC1α, a coactivator central to mitochondrial biogenesis (52, 53). While original reports of PGC1α null mice showed reduced thermogenic capacity, recent adipocyte-specific ablation of PGC1α resulted in only mild cold intolerance and insulin resistance (54, 55). On the other hand, ablation of a PGC1α-interacting partner, IRF4, led to a more severe defect in thermogenesis and energy expenditure (56). The role that IRF4 plays in BAT development is currently unclear, since IRF4 has also been shown to repress adipogenesis as well as expression of general adipocyte markers including PPARγ and FABP4 (57).

**Figure 2:**
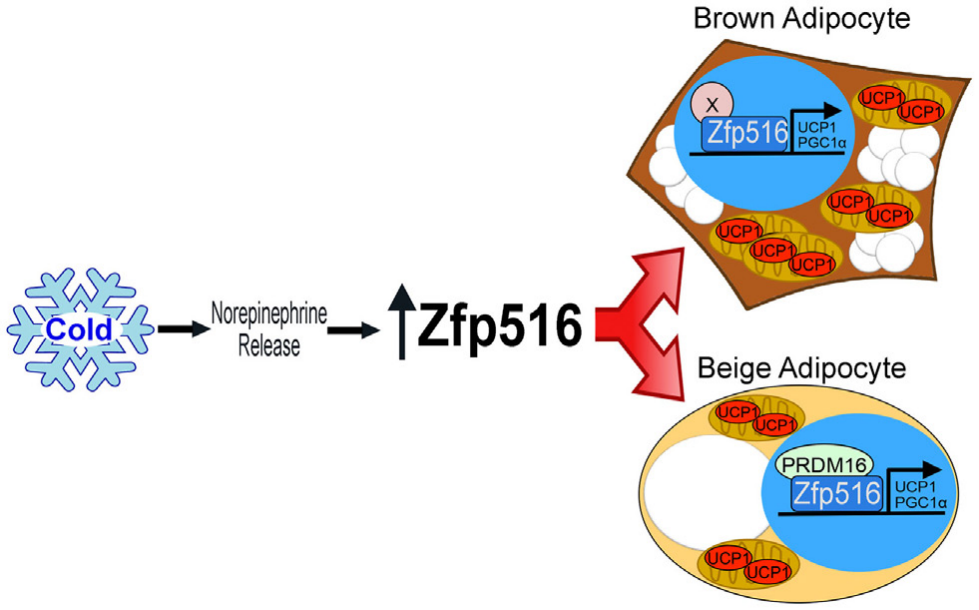
**Regulation of thermogenic adipocytes by Zfp516**. Cold exposure leads to increased Zfp516 levels resulting in the induction of thermogenic genes in beige and brown adipocytes.

Recently, by high throughput screening using the proximal −5.5 kb of the UCP1 promoter, Dempersmier et al. identified the previously uncharacterized transcription factor, Zfp516, as a BAT-enriched, cold-inducible regulator critical for expression of BAT genes and BAT development, as well as browning of iWAT. Ablation of Zfp516 led to impaired BAT development with no detectable UCP1 expression. Conversely, overexpression of Zfp516 in aP2-Zfp516 transgenic mice showed a drastic browning of iWAT with an over 80% increase in tissue oxygen consumption rate. Moreover, overexpression of Zfp516 also resulted in prevention of diet-induced obesity and improved glucose tolerance and insulin sensitivity. Furthermore, ectopic expression of Zfp516 was sufficient to drive C2C12 myoblasts to becoming brown adipocytes (58). As a BAT-enriched, sympathetically regulated transcription factor, Zfp516 may prove to be a central regulator of BAT development and browning of iWAT and thus for non-shivering thermogenesis (Figure 2).

## Signaling in BAT: A Convergence of Signals and Outputs

As an energetically expensive process, thermogenesis in brown and beige adipocytes is under tight hormonal and environmental control. Outside of sympathetic regulation of BAT and browning of iWAT, early work by Tseng et al. identified BMP7 as a critical regulator of BAT development. Ablation of either BMP7 or its receptor led to severely blunted BAT development and impaired thermogenesis (12, 59). While the source of BMP7 driving BAT development is unclear, many tissues, including heart (natriuretic peptides), muscle (Irisin), liver (FGF21), hypothalamus (Bmp8b), thyroid (T4), blood vessels (VEGF), and alternatively activated macrophages (norepinephrine), have been found to signal BAT and WAT to promote thermogenesis and browning (34, 60, 61). Recent work has also highlighted that signaling from brown adipocytes itself can play a role in thermogenesis. Gnad et al. reported that adenosine released from sympathetic nerves as well as from BAT following the release of norepinephrine, to increase the response to norepinephrine through the G_s_-coupled A_2A_ receptor in BAT. Thus, adenosine was shown to act as a feed forward loop, increasing cAMP levels to further activate thermogenic genes (62). Ablation of A_2A_ receptor resulted in an attenuated sympathetic signaling and oxygen consumption in response to cold or β-agonist treatment. However, it has not been shown whether adenosine signaling is required for browning in mice. Regardless, relevant for potential therapeutics, adenosine has already been shown to increase lipolysis and UCP1 expression in human BAT.

Brown adipose tissue-released signaling molecules can also play a role in other tissues. Wang et al. identified Nrg4 as a BAT-enriched secreted protein. Nrg4 was reported to primarily signal through the liver, where it downregulated a panel of genes involved in *de novo* lipogenesis, including *Srebp1c*, *Acc1*, *Scd1*, and *Fasn*. While not affecting thermogenesis, Nrg4 was shown to decrease insulin resistance and hepatosteatosis via gain-of- and loss-of-function studies in mice (63). However, the physiological importance and possible nutritional response of Nrg4 have not yet been assessed. Perhaps, Nrg4 expression inhibits *de novo* lipogenesis to conserve fuels for BAT in times of cold challenge. In addition to Nrg4, other potential BAT-enriched secreted factors that may affect insulin resistance and metabolic homeostasis need to be identified and studied.

## Conclusion and Future Outlook

The affirmation of the existence BAT in human adults has led to an explosion of new information, highlighting the therapeutic potential of BAT. Work on human cold training has shown strong evidence that BAT is prevalent, present in almost all humans to varying amounts, and may be potentially useful in treatment of obesity and type-2 diabetes. However, the inconvenience of cold training makes the “magic pill” to activating hBAT is still the Holy Grail in the field. Understanding the regulation of thermogenesis is, therefore, essential for development of novel therapeutics. Indeed, further work on hormonal and transcriptional regulation of beige/brite and brown adipose development may lead to new insights and therapeutic targets in the future.

## Conflict of Interest Statement

The authors declare that the research was conducted in the absence of any commercial or financial relationships that could be construed as a potential conflict of interest.
